# Potential key genes involved in metabolic resistance to malathion in the southern house mosquito, *Culex quinquefasciatus*, and functional validation of *CYP325BC1* and *CYP9M12* as candidate genes using RNA interference

**DOI:** 10.1186/s12864-023-09241-4

**Published:** 2023-03-29

**Authors:** Xinyue Huang, Phillip E. Kaufman, Giridhar N. Athrey, Chris Fredregill, Christina Alvarez, Vinaya Shetty, Michel A. Slotman

**Affiliations:** 1 Department of Entomology, Texas A&M University Minnie Bell Heep Center, TAMU 2475 370 Olsen Blvd College Station, College Station, TX 77843 USA; 2 Department of Poultry Science, Texas A&M University, College Station, TX 77843 USA; 3 Harris County Public Health, Mosquito & Vector Control Division, Houston, TX 77021 USA

**Keywords:** Metabolic resistance, *Culex quinquefasciatus*, Malathion, Cytochrome P450, Transcriptome, RNA interference

## Abstract

**Background:**

Metabolic detoxification is one of the major mechanisms contributing to the development of resistance in mosquitoes, including the southern house mosquito, *Culex quinquefasciatus*. The three major detoxification supergene families, cytochrome P450s, glutathione S-transferases and general esterases, have been demonstrated to play an important role in metabolic resistance. In this study, we performed differential gene expression analysis based on high-throughput transcriptome sequencing on samples from four experimental groups to give insight into key genes involved in metabolic resistance to malathion in *Cx. quinquefasciatus*. We conducted a whole transcriptome analysis of field captured wild *Cx. quinquefasciatus* from Harris County (WI), Texas and a malathion susceptible laboratory-maintained Sebring colony (CO) to investigate metabolic insecticide resistance. Field captured mosquitoes were also phenotypically classified into the malathion resistant and malathion susceptible groups following a mortality response measure conducted using a Centers for Disease Control and Prevention (CDC) bottle assay. The live (MR) and dead (MS) specimens from the bottle assay, along with an unselected WI sample and a CO sample were processed for total RNA extraction and subjected to whole-transcriptome sequencing.

**Results:**

We demonstrated that the genes coding for detoxification enzymes, particularly cytochrome P450s, were highly up-regulated in the MR group compared to the MS group with similar up-regulation observed in the WI group compared to the CO group. A total of 1,438 genes were differentially expressed in comparison between MR and MS group, including 614 up-regulated genes and 824 down-regulated genes. Additionally, 1,871 genes were differentially expressed in comparison between WI and CO group, including 1,083 up-regulated genes and 788 down-regulated genes. Further analysis on differentially expressed genes from three major detoxification supergene families in both comparisons resulted in 16 detoxification genes as candidates potentially associated with metabolic resistance to malathion. Knockdown of *CYP325BC1* and *CYP9M12* using RNA interference on the laboratory-maintained Sebring strain significantly increased the mortality of *Cx. quinquefasciatus* after exposure to malathion.

**Conclusion:**

We generated substantial transcriptomic evidence on metabolic detoxification of malathion in *Cx. quinquefasciatus*. We also validated the functional roles of two candidate P450 genes identified through DGE analysis. Our results are the first to demonstrate that knockdown of *CYP325BC1* and *CYP9M12* both significantly increased malathion susceptibility in *Cx. quinquefasciatus*, indicating involvement of these two genes in metabolic resistance to malathion.

**Supplementary Information:**

The online version contains supplementary material available at 10.1186/s12864-023-09241-4.

## Background

*Culex quinquefasciatus* currently occurs throughout tropical and temperate regions between 42° N to 42° S [1], is a principal vector of several arboviruses, such as West Nile virus, Japanese encephalitis virus, St. Louis encephalitis virus and Rift Valley fever virus, and is a vector of many filarial parasites [2]. This species acts as a critical bridge vector that can utilize a variety of birds and mammals as hosts, and links reservoir or amplifier hosts to humans in urban, suburban, and rural areas [2]. Due to its ecological plasticity and high reproductive potential, *Cx. quinquefasciatus* rapidly adapts to new areas. The successful establishment of this invasive species is accompanied by increased risk of introduction of pathogens and therefore it continues to be of wide concern [3].

Insecticide-based vector management remains one of the most important strategies to prevent and control the transmission of mosquito-borne diseases. However, the rapid spread of insecticide resistance worldwide in different mosquito species, including *Cx. quinquefasciatus*, has become a threat to successes achieved in disease prevention [4]. Malathion is a widely-used organophosphate mosquito adulticide and is applied through ground-based ultra-low volume applications by Harris County Public Health (HCPH) in areas with known West Nile virus activity [5]. Previous studies demonstrated that *Cx. quinquefasciatus* populations from the Houston, Texas area have a high frequency of resistant individuals with a broad distribution and high levels of resistance to malathion within these resistant individuals [6].

The two major mechanisms for insecticide resistance are target site insensitivity and metabolic resistance. There are a limited number of target sites for neurotoxic insecticides, including the voltage-gated sodium channels (VGSC, or Na_v_ channels), the acetylcholinesterase (AChE1) and the γ-aminobutyric acid receptor (GABA receptor) [7]. Alteration on these target sites can result in resistance to insecticides, including the well-known knockdown resistance (*kdr*) to dichlorodiphenyltrichloroethane (DDT) and pyrethroids. Metabolic resistance, also known as metabolic or enzymatic detoxification, contributes to resistance expression for multiple insecticides. Overproduction of detoxification enzymes usually involves complex expression and regulation of diverse genes and is therefore less tractable than target site modifications [8]. Overexpression of detoxification genes is triggered by several mechanisms, such as copy number variation [9], or regulation of detoxification genes by cis- and trans-regulatory elements [10]. Three major detoxification supergene families, cytochrome P450s, glutathione S-transferases (GSTs), and general esterases, are associated with metabolic resistance in a variety of mosquito species including *Cx. quinquefasciatus* [11].

Associations between permethrin resistance and overexpression of cytochrome P450 genes, such as *CYP9M10*, has been widely reported in *Cx. quinquefasciatus* [12] and validated by multiple methods, including metabolism assays [13] and gene silencing through the TALEN and CRISPR/Cas9 technologies [14]. Previous copy number variation analysis on *Cx. quinquefasciatus* detected an increased copy number of six GST genes in malathion resistant individuals compared to susceptible individuals [15]. More specifically, one of these GST genes, CPIJ018630 (*GSTE2*), showed evidence of amplification from CoNIFER (Copy Number Inference From Exome Reads) results. Increased esterase activity was reported together with observation of amplified esterases Estβ1 and Estα2Estβ2. Mosquitoes with the amplified esterases showed high resistance to organophosphates, with local selection effects determined by insecticide use, indicating the important role of metabolic detoxification in the evolution of malathion resistance within *Cx. quinquefasciatus* populations in Harris County, Texas [6].

The development of next-generation sequencing (NGS) techniques, including RNA-Seq, has become a reliable and cost-effective tool for analysis of gene expression patterns [16]. High sensitivity makes RNA-Seq an ideal technique for comparing expression profiles between different experimental groups and detecting expression levels of specific transcripts [17]. RNA interference (RNAi) is a powerful tool utilized to study gene function in a variety of organisms through exogenous introduction of double-stranded RNA (dsRNA) sequences matching a target gene [18]. RNAi can suppress the expression of a targeted gene through inducing sequence-specific degradation of mRNA and avoid changing the genome of studied organisms.

In this study, we compared gene expression patterns, particularly from three major detoxification supergene families, between malathion resistant and malathion susceptible *Cx. quinquefasciatus* selected using the CDC standard bottle assay. Because exposure to insecticides might increase expression of detoxification genes [19], we also compared our bottle-assay subjects to parental field collected *Cx. quinquefasciatus* that were not exposed to malathion and a laboratory-maintained malathion susceptible strain to explore constitutive-overexpression of malathion resistance-related genes. We identified the most promising candidate genes associated with metabolic detoxification of malathion, and further validated the functional role of two candidate P450 genes, *CYP325BC1* and *CYP9M12*.

## Results

### Transcriptome profiling and differential gene expression analysis

We generated more than 9.54 billion 100-bp cDNA reads across four experimental groups. The number of raw reads was 2,440,769,782 in WI group, 2,402,539,764 in CO group, 2,356,391,146 in MR group, and 2,340,606,486 in MS group. After quality filtering using TrimGalore, more than 75% of reads were successfully mapped to the *Cx. quinquefasciatus* genome (version: CpipJ2.4) from Vectorbase, including 86.97% reads in the WI group, 82.10% reads in the CO group, 81.32% reads in the MR group and 78.86% reads in the MS group (Table 1). Overall, the sequenced reads were assembled into a total of 19,016 genes. Count data were generated using featureCounts version for downstream differential gene expression (DGE) analysis.

**Table 1:** Summary statistics of sequencing data for *Culex quinquefasciatus* transcriptome analysis including mapping totals, Q20 and GC percentage

Experimental Group	Read Length (Filtered)	Number of Reads (Merged)	Total read mapped (%)	Q20 (%)^1^	GC (%)^2^
Wild	99.51	1,191,419,679	1,036,230,100 (86.97)	99.47	47.10
Colony	99.50	1,160,253,093	952,622,888 (82.10)	99.48	47.48
Malathion Resistant	99.51	1,208,329,076	982,622,250 (81.32)	99.47	48.43
Malathion Susceptible	99.51	1,210,151,741	954,384,125 (78.86)	99.47	48.43

^1^Q20% = percentage of bases with Phred quality score (Q score) higher than 20

^2^GC% = percentage of G + C in the reads

The transcriptome comparison performed between the WI group and CO group showed 1,083 up-regulated genes and 788 down-regulated genes in the wild group compared to the colony group. The transcriptome comparison performed between the MR group and the MS group showed 614 up-regulated genes and 824 down-regulated genes in the malathion resistant group compared to the malathion susceptible group. Based on the annotation for the *Cx. quinquefasciatus* genome, we identified 240 genes that code for detoxification enzymes from three major superfamilies, including cytochrome P450, glutathione S-transferase (GST) and general esterase.

We focused on differentially expressed genes (DEGs) from the three major detoxification superfamilies with a false discovery rate (FDR < 0.05). A total of 70 such DEGs were identified in comparisons between the WI and CO groups, including 48 from the P450 superfamily, 19 from the GST superfamily, and three from the general esterase superfamily (Supplementary file 1). A total of 42 DEGs were identified in comparisons between MR group and MS group, including 22 from the P450 superfamily, 13 from the GST superfamily, and seven from the general esterase superfamily (Supplementary file 2). DEGs with FDR values less than 0.05 in both transcriptome comparisons (WI group versus CO group, and MR group versus MS group) were identified as gene candidates. Overall, we identified 12 DEGs of interest (Table 2). Notably, genes such as *CYP4J20*, *CYP9AL1*, *CYP9M12*, *CYP325BC1* and peptide methionine sulfoxide reductase (VectorBase ID: CPIJ018565) were significantly up-regulated in the MR and WI groups. After filtering for off-target effects using siRNA-Finder [20], *CYP325BC1* and *CYP9M12* were chosen for functional validation using RNAi.

**Table 2:** Summary for differences in gene expression (DGE) involved in malathion resistance in *Culex quinquefasciatus* (FDR < 0.05 in both comparisons between WI group versus CO group and between MR group versus MS group)

CPIJ annotation^1^	Gene name	Gene location (Sense/ Non-sense)^2^	Log FC^3^ (WI versus CO)^4^	Log FC^3^ (MR versus MS)^4^
CPIJ002663	Glutathione S-transferase 1–1	DS231848: 92,100 − 92,714 (+)	0.495483867	-1.18997411
CPIJ002786	carboxylesterase	DS231858: 39,753 − 45,680 (-)	-0.587412839	1.856624645
CPIJ005204	Peptide methionine sulfoxide reductase msrA	DS231920: 88,341 − 89,385 (-)	-1.390427104	3.475540072
CPIJ006160	Glutathione s-transferase	DS231921: 397,822 − 412,644 (-)	0.968180082	-1.959035654
CPIJ010231	cytochrome P450 *12F9 (CYP12F9)*	DS232091: 529,206–531,135 (-)	-0.271299583	0.84866247
CPIJ010480	cytochrome P450 *4J20 (CYP4J20)*	DS232089: 494,713 − 496,416 (+)	2.187784633	1.047702497
CPIJ012470	cytochrome P450 *9AL1 (CYP9AL1)*	DS232204: 402,930 − 405,046 (+)	2.07877162	1.193657009
CPIJ014219	cytochrome P450 *9M10-de1b (CYP9M10-de1b)*	DS232322: 166,391 − 170,209 (+)	-1.257222031	1.123581855
CPIJ014220	cytochrome P450 *9M12 (CYP9M12)*	DS232322: 170,566 − 172,216 (+)	0.328056599	1.833454196
CPIJ015958	cytochrome P450 *325BC1 (CYP325BC1)*	DS232542: 95,297 − 97,020 (+)	0.852001468	1.373485365
CPIJ018565	Peptide methionine sulfoxide reductase	DS233029: 61,489 − 62,536 (-)	1.300742526	3.308572035
CPIJ018630	Glutathione S-transferase 1–1	DS233036: 40,439 − 41,178 (-)	1.855584899	-1.096468825

^1^CPIJ = *Culex quinquefasciatus* Johannesburg

^2^Gene location, as described in VectorBase, (S/NS) = Sense = “+” and Non-sense = “-” strand

^3^ Log FC = Log Fold change (log_2_FC calculated by edgeR), positive values represent up-regulated gene expression and negative values represent down-regulated gene expression

^4^WI, CO, MR, MS = mosquito strain sub-types representing wild, colony, malathion resistant and malathion susceptible

**Fig. 1:**
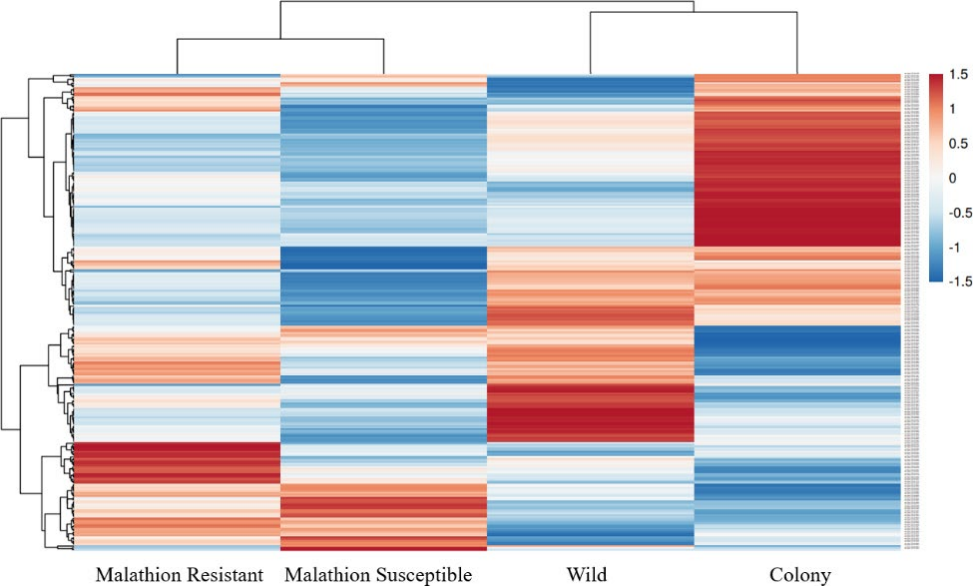
The cluster heatmap was generated by ClustVis that was generated from 240 annotated genes from three major detoxification superfamilies (P450s, GSTs and esterases) to illustrate changes in gene expression. The data are displayed in a grid where each row represents a gene, and each column represents an experimental group. Gene expression levels are color coded, with blue representing down-regulation and red representing up-regulation. Intensity of the color represents the fold change in expression

A cluster heatmap, generated by ClustVis [21], provided a visualization of the expression information of 240 genes from the three major detoxification superfamilies between four experimental groups (Fig. 1). Color intensity between groups corresponds to the difference in the expression of detoxification gene expression in *Cx. quinquefasciatus*.

**Fig. 2:**
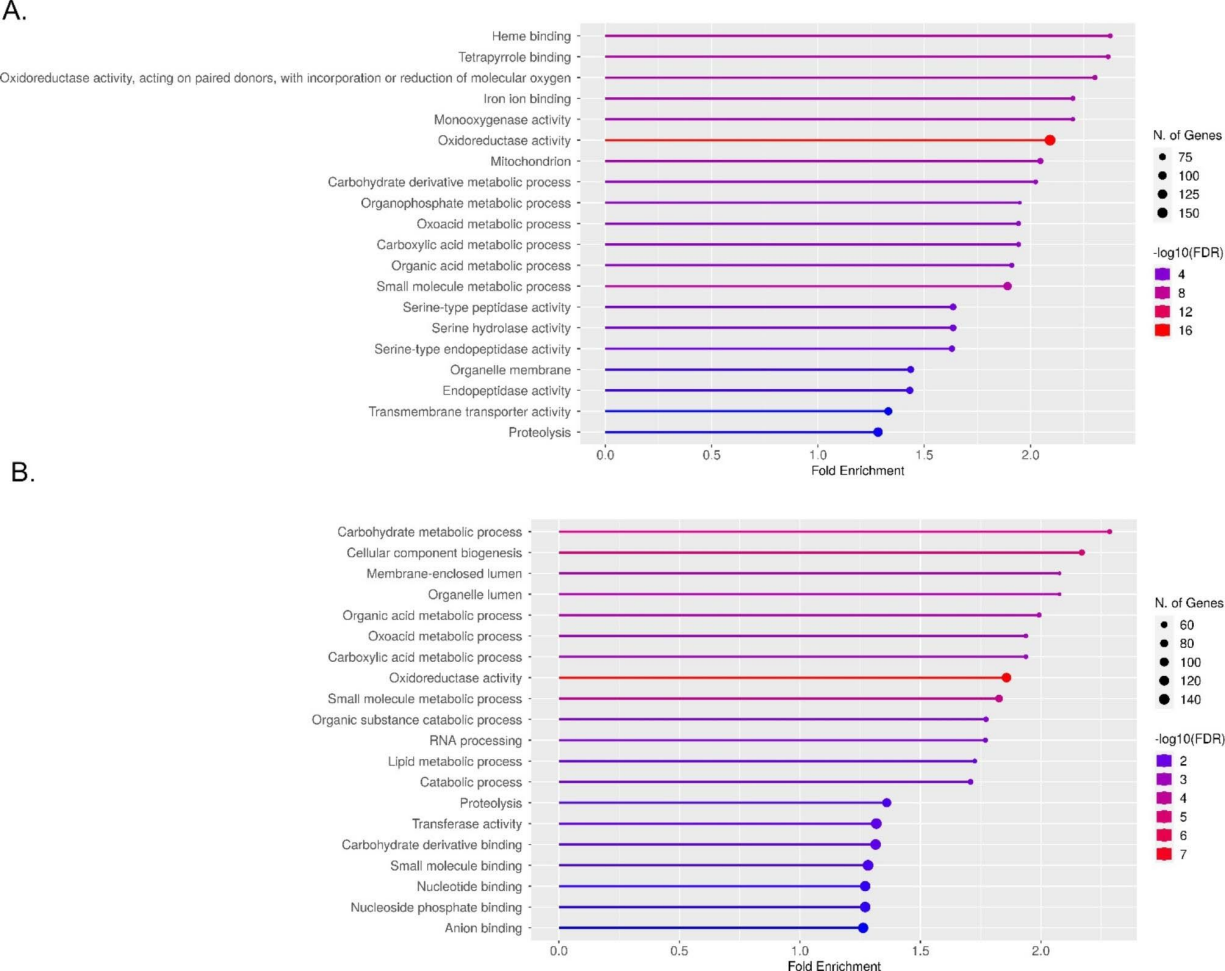
The ShinyGO Pathway Analysis is used to illustrate the top 20 pathways in the comparison performed. All differentially expressed genes (DEGs) with a significant level (FDR value for DEG less than 0.05) are mapped to the referential canonical pathways available in the *Culex quinquefasciatus* database. The size of a circle represents the number of DEGs classified into one specific pathway category. Fold Enrichment (FE) indicates the percentage of DEGs belonging to a pathway, divided by the corresponding percentage in the background. FDR for FE demonstrates how likely the enrichment is by chance. (A) ShinyGO pathway analysis for the top 20 pathways in which the most DEGs (FDR for DEG with a cutoff value of 0.05) in the comparison between *Cx. quinquefasciatus* WI and CO groups were involved. (B) ShinyGO pathway analysis for the top 20 pathways in which the most DEGs (FDR for DEG with a cutoff value of 0.05) in the comparison between *Cx. quinquefasciatus* MR and MS groups were involved

DEGs between WI and CO were categorized into 262 significant pathways (Supplementary file 3) through ShinyGO analysis [22]. In addition, DEGs between MR and MS groups were categorized into a total of 345 significant pathways (Supplementary file 4). Figure 2A and 2B display the top 20 significant pathways sorted by number of DEGs identified in the transcriptome comparisons between WI group versus CO group, and MR group versus MS group, respectively. The top enriched pathway in comparison between WI and CO group is the heme binding pathway, with an FDR value for Fold Enrichment (FE) of 1.23e-7. Interestingly, the organophosphate metabolic process was one of the top 20 significant pathways, with an FDR value for FE of 6.75e-05. The top enriched pathway in comparison between MR and MS group is the carbohydrate metabolic process pathway, with an FDR value for FE of 2.27e-5.

## Double-stranded RNA-mediated RNA interference

**Fig. 3:**
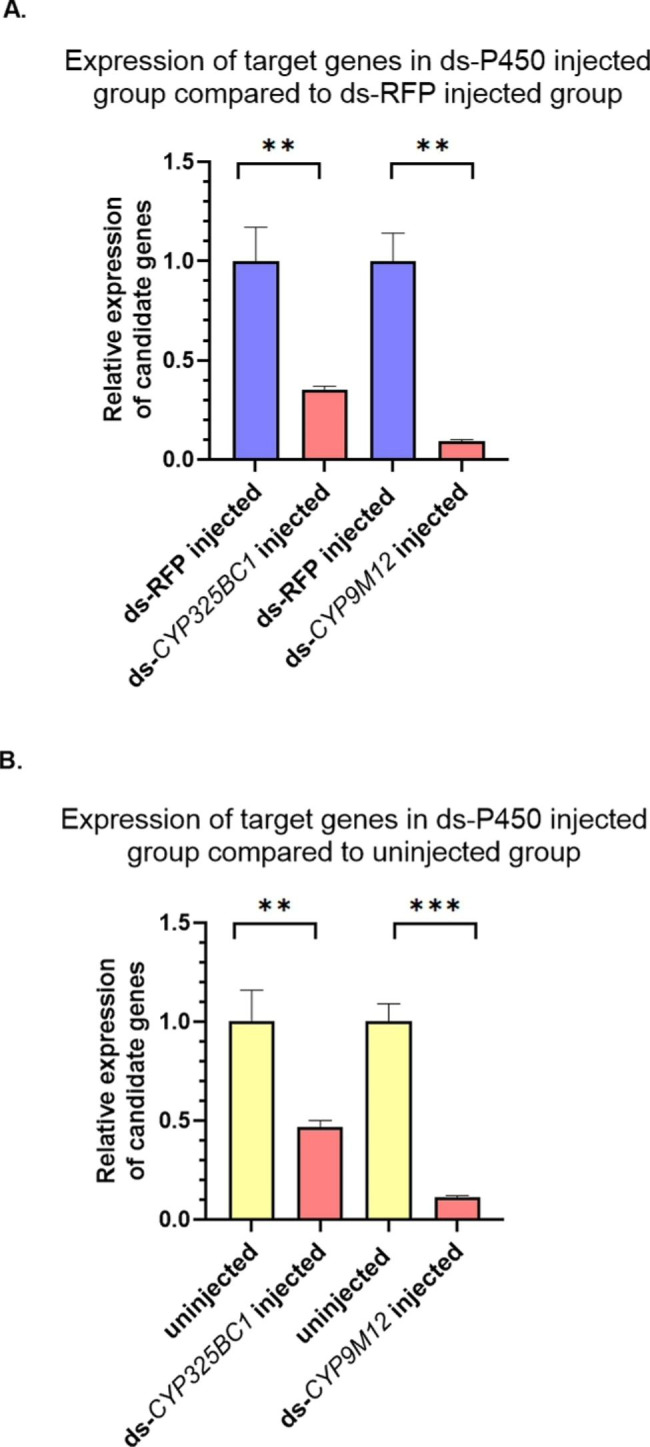
Expression of target genes (mean ± SEM (n ≥ 3)) three days after interference via dsRNA injection detected by real-time quantitative PCR. DsP450s and dsRFP were injected into 3-day-old female adults of *Cx. quinquefasciatus* from the Sebring strain. (A) Relative expression level of target genes, *CYP325BC1* and *CYP9M12*, in dsP450-injected female adults was significantly suppressed compared to dsRFP-injected female adults. (B) Relative expression level of target genes, *CYP325BC1* and *CYP9M12*, in dsP450-injected female adults was significantly suppressed compared to uninjected female adults from the Sebring strain. A Student’s t-test was used for significance analysis. *P < 0.05; **P < 0.01; ***P < 0.001

To validate the functional roles of two candidate P450 genes, *CYP325BC1* and *CYP9M12*, RNAi experiments were conducted in which each P450 gene was knocked down in 3-day-old female adults from the Sebring strain [23]. The RNAi suppression of *CYP325BC1* and *CYP9M12* were conducted in dsP450-injected mosquitoes. The levels detected were 0.35- and 0.09-fold compared to those in dsRFP-injected mosquitoes, respectively (Fig. 3A). RNAi suppressed *CYP325BC1* and *CYP9M12* in dsP450-injected mosquitoes to 0.50- and 0.11-fold compared to those in uninjected mosquitoes from the Sebring strain, respectively (Fig. 3B). These results suggest that the RNAi via dsRNA significantly reduced the expression of *CYP325BC1* and *CYP9M12* in dsP450-injected female adults compared to both dsRFP-injected and uninjected mosquitoes from the Sebring strain.

**Fig. 4:**
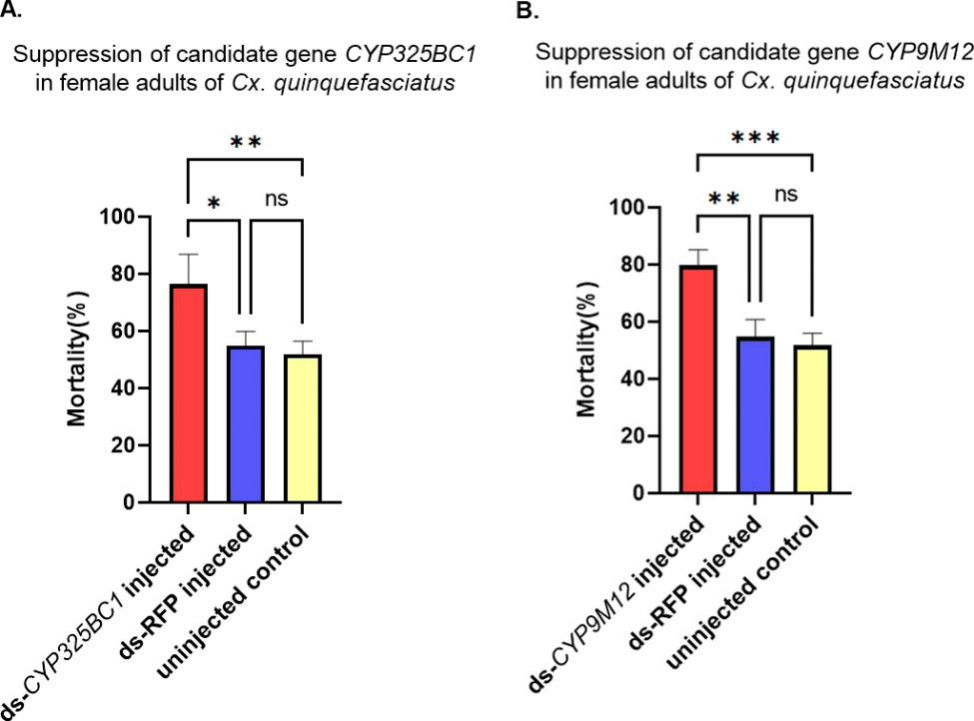
Suppression of candidate P450 genes involved in malathion resistance in *Cx. quinquefasciatus* female adults. The mortality after 120-min exposure to malathion at LC_50_ for dsRNA-injected and uninjected mosquitoes are shown as the mean ± SEM. The Fisher’s LSD test was used for significance analysis. *P < 0.05; **P < 0.01; ***P < 0.001. (A) The mortality of *CYP325BC1* dsRNA-injected female adults is shown in comparison with dsRFP-injected and uninjected female adults from the Sebring strain. The mortality of *CYP325BC1* dsRNA-injected group, dsRFP-injected group and uninjected group is represented as red column, blue column, and green column, respectively. (B) The mortality of *CYP9M12* dsRNA-injected female adults was shown in comparison with dsRFP-injected and uninjected female adults from the Sebring strain

The LC_50_ for malathion in the Sebring strain was 6.727 µg/mL (or µg/bottle, as each bottle contained 1 mL solution in the CDC standard bioassay) with a 95% confidence interval from 5.670 to 7.835 µg/mL. This concentration was subsequently used in a standard bottle assay to assess the impact of RNAi knockdown of P450 genes in *Cx. quinquefasciatus*. After RNAi of two candidate P450 genes, *CYP325BC1* and *CYP9M12*, mosquito mortality was evaluated to determine the relationship between the expression of these candidate genes and malathion tolerance in *Cx. quinquefasciatus*, using the LC_50_ concentration from the preliminary bottle assay. As shown in Fig. 4A, the mean mortality after exposure to the LC_50_ concentration of malathion in the *CYP325BC1* dsRNA-injected group, dsRFP-injected group and uninjected group were 76.39%, 54.85% and 51.85%, respectively. The mortality in the *CYP325BC1* dsRNA-injected group was increased significantly compared to the mortality in the dsRFP-injected group and uninjected group with *p*-values of 0.0107 and 0.0059, respectively. As shown in Fig. 4B, the average mortality after exposure to malathion in the *CYP9M12* dsRNA-injected group, dsRFP-injected group and uninjected group were 79.76%, 54.83% and 51.76%, respectively. The mortality in the *CYP9M12* dsRNA-injected group was significantly greater when compared to the mortality in the dsRFP-injected group and uninjected group with *p*-values of 0.012 and 0.006, respectively.

## Discussion

Insecticide resistance is a threat to effective vector control efforts, particularly with respect to insects that contribute to the spread of human infectious disease [24]. The mechanism of insecticide resistance has been studied extensively in mosquitoes and can be classified into several categories, target site insensitivity, metabolic detoxification, behavioral adaptation, and cuticular modification [25]. Metabolic detoxification refers to resistance due to the overexpression of detoxification genes that results in increased activities of detoxification enzymes, particularly those from the three superfamilies: P450, GST and general esterases.

Next-generation sequencing has become affordable for numerous applications and has been applied for transcriptome analysis and discovery of genes associated with insecticide resistance [15]. Most transcriptome profiling analyses conducted in mosquitoes have focused on genes involved in resistance to pyrethroid insecticides. Additionally, the majority of mosquito studies address the constitutive overexpression of detoxification genes in resistant strains compared to susceptible strains. But induction of xenobiotics, including insecticides, also can result in up-regulated expression of detoxification genes, also known as induced overexpression. Induced overexpression of multiple P450s has been reported in response to pyrethroid exposure in *Cx. quinquefasciatus* [12].

In this study, we explored constitutive overexpression of detoxification genes following malathion exposure by comparing transcriptome profiles of field-collected and laboratory-maintained susceptible *Cx. quinquefasciatus*. We identified 1,871 DEGs (FDR < 0.05) between WI group and CO group. Of these, a total of 70 DEGs were identified from the three major detoxification superfamilies, including 48 from the P450s, 19 from the GSTs, and three from the general esterases. Thereafter, we investigated malathion-induced overexpression of detoxification genes by comparing within population transcriptome profiles of resistant and susceptible *Cx. quinquefasciatus* selected from bottle assay results for malathion. We identified a total of 1,438 DEGs (FDR < 0.05) between the MR group and MS group. Of these, a total of 42 DEGs were identified from the three major detoxification superfamilies, including 22 from the P450s, 13 from the GSTs, and seven from the general esterases. The 12 candidate detoxification genes were found differentially expressed at a significant level (FDR < 0.05), as listed in Table 2. Notably, genes such as *CYP4J20, CYP9AL1, CYP9M12, CYP325BC1* and peptide methionine sulfoxide reductase (VectorBase ID: CPIJ018565) were significantly up-regulated in both transcriptome comparisons.

Several candidate genes have been reported to be associated with metabolic detoxification of insecticides in previous studies on *Cx. quinquefasciatus*. For instance, *CYP9M10* was widely reported to be linked to permethrin resistance [12] and its functional role in detoxification of permethrin was validated by multiple methods, including metabolism [13] and gene silencing through the TALEN and CRISPR/Cas9 technologies [14]. *CYP9AL1* was reported to be the major gene at the center of a resistance regulatory network in the northern house mosquito, *Cx. pipiens pallens*. The involvement of *CYP9AL1* in insecticide resistance in *Cx. pipiens* was validated by siRNA microinjection and a CDC bottle bioassay [26]. Additionally, knockdown of the *CYP9AL1* gene significantly decreased permethrin resistance in *Cx. quinquefasciatus* larvae [27]. The *CYP12F9*, *CYP4J20* and *CYP9M12* genes were reported as down-regulated in a permethrin selected *Cx. quinquefasciatus* population at larval stage [28]. However, in our study we found conflicting results where the expression of *CYP12F9* was lower in the WI group as compared to CO group, but higher in the MR group as compared to the MS group. However, *CYP4J20* and *CYP9M12* were overexpressed in both the WI group and MR group as compared to the more susceptible CO group and MS group, respectively. The *CYP325BC1* gene has been reported to be up-regulated in a permethrin-selected highly resistant HAmCq^G8^ strain [29]. Functional characterization of the *CYP9M12* and *CYP325BC1* genes in *Cx. quinquefasciatus* have not been published prior to this study. The varied expression level of detoxification genes in the different *Cx. quinquefasciatus* strains indicated that the effects of permethrin and malathion insecticide induction may be different. Certain genes or combinations of genes likely play a more important role in development of insecticide resistance, even compared to others from the same superfamilies that also may be overexpressed. To date, the exact number of genes involved in metabolic detoxification of insecticides remains unclear. More efforts are needed to further elucidate regulating mechanisms of candidate detoxification genes.

The ClustVis cluster heatmap (Fig. 1) provided an overall vision on the detoxification gene expression profiles of the four strains under specific circumstances. Samples from the MR and MS groups represented the physiological condition of alive and dead WI mosquitoes after exposure to malathion, respectively. The heatmap illustrates a great portion of overlapping genes overexpressed in both the MR and MS groups, but not in the WI and CO groups, indicating a xenobiotic-induced mode to regulate detoxification genes in *Cx. quinquefasciatus*. The susceptible CO group demonstrated a different expression pattern as compared to the WI group sampled from a mosquito population in Harris County, Texas, indicating a constitutive mode involved in regulation of detoxification genes. Like other insects, which are suggested to be able to utilize both the induced mode and constitutive mode to regulate detoxification gene expression [30], these two modes may be simultaneously employed in overexpression of detoxification genes associated with malathion resistance in *Cx. quinquefasciatus* [31].

ShinyGO enrichment analysis can integrate genetic and systemic functional information from databases such as gene ontology (GO) and provide interpretation for large-scale data sets generated by high-throughput sequencing technologies [22]. We performed the ShinyGO enrichment analysis with all the detected DEGs using a cutoff FDR value of 0.05. From this analysis, the top 20 significant pathway categories that included the largest number of DEGs were listed in Fig. 2. The heme binding category (Term GO: 0020037) was identified with the largest FE value in comparison between WI and CO groups (Fig. 2A). The heme binding category was reported to be an overrepresented GO term linked to pyrethroid resistance in *Aedes aegypti* [32] and *Cx. quinquefasciatus* [29]. These results support the hypothesis that cytochrome P450s, a group of heme-thiolate monooxygenases, are necessary for detoxification of xenobiotics, including multiple classes of insecticides [33]. Importantly, the organophosphate metabolic process category (Term GO: 0019637) also was one of the top 20 significant pathway categories. Considering that malathion is a frequently applied organophosphate insecticide used for vector control efforts in Harris County, constitutive overexpression of genes related with organophosphate metabolic processes is a reasonable finding in wild *Cx. quinquefasciatus*, as compared to the laboratory-maintained susceptible strain. The carbohydrate metabolic process category (Term GO: 0005975) was identified with the largest FE value in comparison between the MR and MS groups (Fig. 2B) and was a significant pathway category in the comparison between the WI and CO groups (Fig. 2A). These results are consistent with our knowledge that carbohydrate esterases play important roles in metabolism of organophosphate insecticides such as malathion [6].

Malathion resistance due to increased expression of P450, GST and general esterase genes can be found in a variety of arthropod species, such as the fruit flies, *Drosophila melanogaster* [34, 35] and *Bactrocera dorsalis* [36, 37]. RNAi was employed to determine the metabolic roles of GSTs to malathion tolerance in *B. dorsalis* [36]. As for *Cx. quinquefasciatus*, overexpression of P450 genes have been widely identified in different insecticide resistant strains. Overexpression of specific P450 genes, such as *CYP9M10*, was reported in *Cx. quinquefasciatus* resistant strains collected from various geographic regions such as Saudi Arabia [38], United States [13], Kenya, Singapore, and Vietnam [39]. A study on *Cx. quinquefasciatus* reported that two P450 genes (*CYP4C52V1* and *CYP6AA7*) conferred permethrin resistance and subsequently demonstrated their functional roles in resistance expression using RNAi technology [27]. They also conducted a parallel RNAi experiment with *CYP6BY3*, a non-overexpressed gene, and found no effect on insecticide resistance after successful knockdown of this control P450 gene.

In our study, RNAi was utilized to evaluate the functional roles of two candidate P450 genes, *CYP325BC1* and *CYP9M12*, which were found overexpressed in malathion resistant *Cx. quinquefasciatus*. Known off-target effects in RNAi indicated that knockdown of other genes also may alter the susceptibility of the mosquitoes [40]. The length of contiguous matching bases and partial sequence identity is a key factor determining the off-target effects of dsRNA-induced RNAi [41]. To avoid off-target impacts, we conducted off-target effect prediction using a software package, siRNA-Finder (si-Fi), prior to designing our dsRNA. Through these efforts we did not observe off-target effects. Our RNAi approach successfully decreased the expression level of *CYP325BC1* and *CYP9M12*, the two targeted P450 genes (Fig. 3) and correspondingly increased the mortality rate after exposure to malathion (Fig. 4). Our findings demonstrated that the reduction of transcript expression of these P450 genes was associated with increased susceptibility to malathion in the susceptible Sebring strain, supporting their role in the tolerance of *Cx. quinquefasciatus* to malathion.

Taken together, our results suggest that metabolic detoxification contributes greatly to malathion insecticide tolerance in *Cx. quinquefasciatus* in Harris County. Genes from detoxification superfamilies, such as P450, GST and general esterases, were indicated to be involved in metabolization of xenobiotics. At the same time, the functional role of each specific gene can be varied and regulated accordingly. Some highlighted candidate genes may play major roles in the detoxification process. Here we presented a complete pipeline to filter candidate detoxification genes based on an RNA-Seq technique and a downstream DGE analysis. The reliability of transcriptome profiling and core gene identification was validated by the data demonstrating that knockdown of *CYP325BC1* and *CYP9M12* using RNAi technology significantly increased mortality of *Cx. quinquefasciatus*.

Although we observed overexpression of several candidate P450 genes and revealed their association with resistance to malathion in *Cx. quinquefasciatus*, the underlying molecular basis for this detoxification process and its regulatory mechanism remains unclear. Additionally, the expression level of detoxification genes, such as P450s and general esterases, can vary with different developmental stages of mosquitoes. A study performed on the highly permethrin resistant strain, HAmCqG8, reported that *CYP9M10* was specifically overexpressed in the fourth instar larval stage compared to the adult stage [42]. Besides, a study on *Anopheles gambiae* indicated that early exposure to sub-lethal dose of permethrin at larval stage can impact the immune response of the adults [43]. Additionally, different long-lasting expression of specific genes in *Aedes aegypti* were reported to be caused by changing larval rearing temperature, which led to different responses to malathion during adult stage [44]. These articles suggested that gene expression pattern in larval stage may differ from that in adult stage; and DGE induced in larval stage may have long-lasting impact even in adult stage. Our study focused on the adult stage of *Cx. quinquefasciatus*; therefore, there is still a need for further investigation on other developmental stages, especially the larval stage, as a countermeasure for development of resistance to larvicides such as malathion, permethrin and temephos [45].

## Conclusion

By identifying differentially expressed genes (DEGs) between untreated *Culex quinquefasciatus* from Harris County and individuals from a laboratory-maintained malathion-susceptible Sebring strain, we determined identified detoxification genes associated with malathion tolerance through constitutive overexpression. Also, by identifying DEGs between phenotypic malathion resistant and susceptible individuals, we determined which detoxification genes contributed to malathion tolerance through overexpression induced by exposure to malathion. Multiple significantly upregulated detoxification genes - *CYP4J20*, *CYP9AL1*, *CYP9M12*, *CYP325BC1* and the peptide methionine sulfoxide reductase (VectorBase ID: CPIJ018565) were hypothesized to play key roles in metabolic detoxification of malathion, and therefore were identified as candidate genes involved in malathion tolerance. To further validate the role of candidate genes identified in malathion tolerance transcriptome profiling of *Cx. quinquefasciatus* in Harris County, we performed double-stranded RNA-mediated RNAi to suppress the expression level *CYP325BC1* and *CYP9M12* in adult females of the Sebring strain. Knockdown of *CYP325BC1* and *CYP9M12* both significantly increased the mortality rate of *Cx. quinquefasciatus* after exposure to malathion.

This research is the first to study detoxification genes associated with malathion tolerance in *Cx. quinquefasciatus* through a combination of transcriptome profiling, DGE analysis and RNAi validation. Here we demonstrated the essential roles of *CYP325BC* and *CYP9M12* genes in malathion tolerance of *Cx. quinquefasciatus.* We successfully used our approach efficiently filtering genes expressed during metabolic detoxification and other important physiological processes in mosquito vectors to direct our study of specific candidate genes likely involved in phenotypic expression of resistance in *Cx. quinquefasciatus*. The knowledge generated here can be utilized for future research into pesticide resistance mechanisms in *Cx. quinquefasciatus*. Our findings provide a direction for exploration on underlying molecular mechanism that regulates metabolic detoxification of malathion associated with *CYP325BC* and *CYP9M12* genes. Homology modeling and docking analysis, an in-silico structure-based prediction technique can be performed to study the structural and functional interactions between malathion and cytochrome P450 monooxygenases coded by these candidate genes [46]. Finally, the approach demonstrated here can serve as a suitable model for similar studies in other mosquito vectors.

## Materials and methods

### Transcriptome profiling and differential gene expression analysis

There were four experimental groups included in the transcriptome profiling study, Wild group (WI), Colony group (CO), Malathion Resistant group (MR) and Malathion Susceptible group (MS). Field-collected *Cx. quinquefasciatus* mosquitoes (WI) from Harris County, Texas were phenotyped for susceptibility to malathion and were determined as resistant (MR) or susceptible to malathion (MS) based on results from the CDC standard bottle assay. The Colony group (CO) was sourced from an insecticide susceptible Sebring colony strain maintained by the Mosquito and Vector Control Division of HCPH insectary that was reared under the same conditions without exposure to insecticide. The Sebring strain was originally collected from Sebring, Florida in 1988 and has been maintained by CDC in Fort Collins since 2004 [23].

The Wild group (WI) *Cx. quinquefasciatus* samples consisted of eggs collected in Houston, Texas, USA. Collected eggs were reared to adults in the insectary at 26 ± 1 ℃. Three days after emergence, female WI adults were knocked down in a refrigerator at -20 ℃ for 30 min, then transferred individually into a 5 mL tube, and stored in 200 µL prechilled Invitrogen™ RNA*later™*-ICE Frozen Tissue Transition Solution (Thermo Fisher Scientific, Carlsbad, California) at 4 ℃ overnight and then at -80 ℃ for long-term storage. The Colony group (CO) was an insecticide susceptible Sebring colony strain reared under the same conditions without exposure to insecticide in the HCPH insectary. Female CO mosquitoes were knocked down three days after emergence and stored in RNA*later™*-ICE solution at 4 ℃ overnight and then at -80 ℃ for long-term storage, as described for the WI colony. Additional field captured *Cx. quinquefasciatus* were subjected to the CDC standard bottle bioassay following instructions in the CONUS procedure where 15–30 three-day-old females were exposed to malathion (400 µg/bottle) in 250-mL bottles. Mosquitoes that were dead at the end of a 120 min exposure period were transferred into RNA*later™*-ICE solution and labeled as Malathion Susceptible (MS) group. Surviving mosquitoes were held for 24 h under standard insectary conditions. After this holding period, mosquitoes still alive were labeled as Malathion Resistant (MR) group. Then these mosquitoes were transferred into RNAlater™-ICE solution and stored at 4 ℃ overnight and then at -80 ℃ for long-term storage before species diagnostic and RNA extraction.

Before RNA extraction, a species diagnostic PCR for each sample was conducted to verify the morphological identification. The PCR reactions were prepared with Thermo Scientific™ Phire Tissue Direct PCR Master Mix (Thermo Fisher Scientific, Carlsbad, California) using a *Culex pipiens* complex-specific primer set, including *CP16* and *PQ10*, described by Crabtree et al. [47]. *CP16* is complementary to 28s rDNA gene and *PQ10* is *Culex pipiens* complex-specific. The reaction using this pair of primers amplifies a 698-bp fragment from specimens in the *Cx. pipiens* complex. One leg of each mosquito individual was tested to determine species identity and remaining body parts of females were held in RNAlater™-ICE solution for RNA extraction. For each leg tested, a 20 µL PCR reaction was generated which included 10 µL 2× PCR master mix mentioned above, 0.5 µM *CP16* primer, 0.5 µM *PQ10* primer, one mosquito leg and nuclease-free water. The amplification program had an initial denaturation cycle for 5 min at 98 ℃, followed with 40 cycles performed for 5 s at 98 ℃; 5 s at 55 ℃; and 20 s at 72 ℃, and a final extension cycle for 1 min at 72 ℃. PCR amplification products were then loaded to the wells of 2% agarose gel covered with 1× TAE buffer. The electrophoresis process lasted for 20 min at 125 Volts. Gel imaging was obtained from the Invitrogen™ iBright 1500 system (Thermo Fisher Scientific, Carlsbad, California). An Invitrogen™ 100 bp DNA Ladder (Thermo Fisher Scientific, Carlsbad, California) was run with each batch of samples to further distinguish *Cx. quinquefasciatus*. Samples that showed a clear 698-bp band were used for RNA extraction in following step. Between 68 and 83 mosquitoes in each pool were screened by this PCR protocol, with no more than two mosquitoes failing to produce the desired *Cx. pipiens*-complex band (success rate of > 97.5%). Within each set of samples producing the band, 50 specimens were used in the RNA extraction process.

To identify genes important in malathion resistance of *Cx. quinquefasciatus* by gene expression profiling through RNA sequencing, mosquitoes from four experimental groups described above were subjected to total RNA extractions. We had four experimental groups in total and five technicalreplicates were included in each experimental group. RNAlater™-ICE solution was removed from the mosquito samples and ten mosquito individuals were pooled and processed as one replicate. Mosquito tissues were transferred into a 2 mL tube and 1 mL QIAzol Lysis Reagent (Qiagen, Germantown, Maryland) was added per 100 mg tissue. Lysis and homogenization were conducted with a TissueRuptor® (Qiagen, Germantown, Maryland). The protocol used was developed for fatty tissues using QIAzol Lysis Reagent. RNA products were purified with the QIAGEN® RNeasy Mini Kit (Qiagen, Germantown, Maryland). A primary quantification for RNA was performed with Invitrogen™ Qubit Fluorometer (Thermo Fisher Scientific, Carlsbad, California) before RNA samples were submitted to the Texas A&M University Genomics Facility (TxGen). Initially, the samples were analyzed on the Agilent 2100 bioanalyzer (Model Agilent Technologies, Santa Clara, California) for quality control. After that, cDNA libraries were prepared for sequencing, running on an Illumina® NovaSeq™ 6000 S2 system (Illumina, San Diego, California) with PerkinElmer NEXTFLEX® Rapid Directional RNA-Seq Kit (PerkinElmer, Waltham, Massachusetts). For each replicate, a NovaSeq 6000 S2 2 × 100 bp v1.5 flow cell was used. Samples were run simultaneously on one flow cell usingusing two lanes.

RNA sequencing data in fastq format was downloaded from the TxGen website [48]. The analysis pipeline was developed based on the Genome Analysis Toolkit (GATK) Best Practices for RNA sequencing data [49]. Adaptors were trimmed with TrimGalore version 0.6.4_dev [50]. After trimming and quality control, reads were mapped to the whole genome sequences of *Cx. quinquefasciatus* using STAR version 2.7.3a [51]. *Culex quinquefasciatus* genome data were downloaded from VectorBase [52] and the reference genome construction version was CpipJ2.4. The reference genome was indexed before a two-pass alignment step. To improve sensitivity and accuracy, the output of a first alignment pass was used for generating annotation for a second alignment pass. Next, Picard tools version 2.20.1 [53] was used to identify and add Read Group (RG) for output from second alignment. Read count data were generated using featureCounts version 1.6.0 [54].

Differential gene expression (DGE) data analysis was completed within R version 4.1.1 [55]. DGE analysis was conducted by edgeR version 3.36.0 [56]. Trimmed Mean of M-values (TMM) value [56] employed in edgeR was applied to normalize raw count data. Low-count data were filtered and a calculation for normalization factors was utilized to improve comparison accuracy. A cluster heatmap was generated by ClustVis to visualize the expression information of differentially expressed genes (DEGs) from three major detoxification superfamilies across four experimental groups [21].

GO enrichment analysis was performed with DGE data obtained in the last step using ShinyGO version 0.76 [22]. ShinyGO is a graphical tool for enrichment analysis based on annotation databases acquired from Ensembl and STRING-db, which can link gene lists with molecular pathways and functional categories. DEGs with a false discovery rate (FDR for DEG) cutoff value of 0.05 detected in previous steps were imported for an insight into their functional classifications. FE values were calculated with a false discovery rate (FDR for FE) from the percentage of DEGs belonging to a pathway, divided by the corresponding percentage of genes in the background. FE was an important metric describing how drastically DEGs within a certain pathway were overrepresented.

## Double-stranded RNA-mediated RNA interference

The laboratory-maintained malathion susceptible Sebring strain *Cx. quinquefasciatus* colony was used for RNAi in this research to validate the association between malathion detoxification and the detected overexpression of candidate resistance genes *CYP9M12* and *CYP325BC1*, identified in transcriptome profiling. Eggs of the Sebring strain were obtained from HCPH. Mosquito larvae were fed with crushed fish food and maintained in the incubator at a temperature of 28 ℃ and 75 ± 5% relative humidity (RH), under a 12:12 h light:dark (L:D) photoperiod. The adult mosquitoes were maintained under standard insectary conditions at 26 ± 1 ℃ and 75 ± 5% RH, under a 12:12 h L:D photoperiod. Adult mosquitoes were fed with 10% sugar water prior to blood-feeding five days after emergence. If no eggs were collected, a second blood feeding was provided three days after the first feeding. Sheep blood was provided and maintained at 37 ℃ via a hot-water cycling temperature control device for four hours during each blood-feeding event. *Cx. quinquefasciatus* from the third generation of this colony were used for qRT-PCR and individuals from the fourth generation were used for RNAi.

According to the results from transcriptome profiling, the expression of two P450 genes, *CYP9M12* and *CYP325BC1*, were identified as being up-regulated in the MR group and the WI group in comparison to the MS group and the CO group, respectively. RNAi techniques were utilized to suppress the expression of these targeted P450 genes to verify their function and association with malathion resistance. The double-stranded RNA of a red fluorescent protein (ds-RFP), *DsRed1*, was also synthesized and injected to control for a potential false-positive result due to the injection effect.

Genomic DNA of *Cx. quinquefasciatus* and cloned plasmid DNA were used for the two sets of amplification PCRs to generate DNA templates for transcription of the sense and antisense RNA. Genomic DNA of *Cx. quinquefasciatus* was used for the dsRNA synthesis of *CYP9M12* gene and *CYP325BC1* gene, while cloned plasmid DNA was used for the dsRNA synthesis of *DsRed1* gene. The amplification primer sequences were designed using the Primer3 program version 0.4.0 [57] with a length between 20 to 24 nt and an optimum melting temperature (T_m_) at 60 ℃. After choosing the appropriate primer sequence, the T7 promoter sequence (TAATACGACTCACTATAGGG) was added to the 5’ end of both primers (Table 3). The amplification PCR was conducted using Invitrogen™ Platinum™ II Taq Hot-Start Kit (Thermo Fisher Scientific, Carlsbad, California). Each 50 µL amplification PCR reaction included 10 µL 5× PCR buffer, 1 µL dNTP mix (10 mM), 1 µL forward and reverse primers (1 µM), 0.4 µL Taq DNA polymerase, 33.6 µL nuclease-free water and 1 µL genomic DNA or plasmid DNA (no more than 500 ng). The amplification PCR program started with a cycle for 2 min at 94 ℃, followed with 35 cycles performed for 45 s at 94 ℃; 45 s at 56 ℃; and 60 s at 72 ℃, and a final cycle for 5 min at 72 ℃. 5 µL of the amplification products were loaded into a 1% agarose gel covered with 1× TAE buffer. Gels were run for 20 min at 125 volts on the Galileo Bioscience horizontal system (Galileo Bioscience, Stoneham, Massachusetts). The electrophoresis gel was visualized and analyzed using an Invitrogen™ iBright™ FL1500 Imager System (Thermo Fisher Scientific, Carlsbad, California). Amplified DNA products were purified with NucleoSpin® Gel and PCR Clean-up Kits (Takara Bio, San Jose, California). The purification process was completed following the manufacturer’s protocol with optimization on the final elution step, in which 20uL of Buffer NE was added to the column and incubated for 3 min at room temperature. After this step, the purified products were verified using electrophoresis analysis with a 2% agarose gel.

**Table 3:** The information of primers, including VectorBase ID, gene name, primer use and sequences. Primers were used for PCR amplification of dsRNA template DNA, in vitro transcription of dsRNA from cDNA and silencing validation by real-time quantitative reverse transcription PCR (qRT-PCR) for RNAi validation of candidate P450 genes involved in malathion resistance of *Culex quinquefasciatus*. Each primer set includes a forward sequence (F) and a reverse sequence (R). An additional probe (P) sequence was used for probe-based qRT-PCR.

VectorBase ID	Gene Name	Primer Use	Primer sequences (5′–3′)
CPIJ014220	cytochrome P450 *CYP9M12*	amplification	F: CCTTTTTCTTCGGAGGTATCG
			R: AAAAGCCGTTCTTCGCACT
		dsRNA from cDNA	F: TAATACGACTCACTATAGGGCCATGTTGTGCTTTGCAATC
			R: TAATACGACTCACTATAGGGGTTCCGAAATCTCCACCGTA
		qRT-PCR	F: GTTACTGTGAGGGGGCAATG
			P: AATCGGCTGAGGGGAGGATC
			R: CATGCCAAACGAGATTGATG
CPIJ015958	cytochrome P450 *CYP325BC1*	amplification	F: GGATATGATCTGCGCAACTG
			R: CGGCAAATATGATTTCATCCA
		dsRNA from cDNA	F: TAATACGACTCACTATAGGGGGGAGTGGACATAAACGTGC
			R: TAATACGACTCACTATAGGGAATGTTCCGCTATGGATTCG
		qRT-PCR	F: AAGTCGGAGGACTGTCTGGA
			P: TCATCCTGGAAAGCGGTTCG
			R: GCTTGGAGACTTGCTCAACC
CPIJ000162	60 S ribosomal protein L8	qRT-PCR	F: ACTTCCGTGACCCGTACAAG
			P: AGCTCCGCAAGCAGCTGTTC
			R: ACCGGTCTTTTCCTCCAGAT
	*DsRed1*	amplification	F: CGATGGTGTAGTCCTCGTTG
			R: GCTCCTCCAAGAACGTCATC
		dsRNA from cDNA	F: TAATACGACTCACTATAGGGGGTGTAGTCCTCGTTGTGG
			R: TAATACGACTCACTATAGGGGAAGCTGAAGGTGACCAAGG
		qRT-PCR	F: TAGTCCTCGTTGTGGGAGGT
			P: ACGGGCTTCTTGGCCATGTA
			R: CCCCGTAATGCAGAAGAAGA

In vitro transcription was performed using an Invitrogen™ MEGAscript™ T7 Transcription Kit (Thermo Fisher Scientific, Carlsbad, California) in a dedicated area treated with Invitrogen™ RNase*Zap*™ to be free of RNase contamination. Each transcription reaction included 2 µL ATP, 2 µL CTP, 2 µL GTP, 2 µL UTP, 2 µL 10× Reaction Buffer, 2 µL T7 Enzyme Mix, and 8 µL purified DNA template. The reaction mixture was incubated overnight (up to 16 h) at 37 ℃ in the thermocycler. After incubation, RNA products were treated with 0.5 µL of DNase enzyme provided in the MEGAscript™ T7 Transcription Kit and incubated at 37 ℃ for 20 min in the thermocycler. Next, DNase-treated RNA products were purified with MEGAclear™ Transcription Clean-Up Kit (Thermo Fisher Scientific, Carlsbad, California) following a modified manufacturer’s protocol. Pelleted RNA was dissolved in 20 µL of RNase-free water. The concentration of purified RNA products was quantified using BioTek™ Epoch™ Microplate Spectrophotometer (BioTek, Winooski, Vermont).

Finally, dsRNA was produced from the sense and antisense RNA strands in the annealing step. The 100 µL annealing mix included 5 µL sense RNA, 5 µL antisense RNA, 10 µL 10× annealing buffer and 80 µL nuclease-free water. The annealing mix was heated at 95℃ for 1 min and then incubated at room temperature overnight. 10 µL 3 M sodium acetate (pH 5.2) and 250 µL ethanol were added into the annealing mix to precipitate RNA and the solution was incubated at − 80 ℃ overnight. The precipitate was collected by centrifugation using Eppendorf® Microcentrifuge 5415 C (Eppendorf, Enfield, Connecticut) at maximum speed (14,000 rpm) for 30 min at 4 ℃. The supernatant was discarded, and the pellet was washed with 1 mL pre-chilled 70% ethanol. RNA was recovered by centrifugation at 14,000 rpm for 5 min at 4 ℃. The pellet was dissolved in RNase-free water to yield a 1 µg/µL stock and then stored at − 80 ℃. The integrity of dsRNAs were checked using electrophoresis analysis with 2% agarose gel. Sense and antisense single-stranded RNA (ssRNA) and template DNA in the transcription reaction were used as control.

Total RNA was extracted from the whole body of mosquito samples. Five mosquito individuals were pooled in each replicate. Each experiment was repeated three times with independent biological samples. Real-time quantitative PCR (qRT-PCR) was used to detect the expression level of the target genes and validate the degree of interference after dsRNA injection. The relative expression level of target genes was quantified 3 days after the injection of *CYP325BC1, CYP9M12* and *DsRed1* dsRNAs. The qRT-PCR was conducted using a QuantiFast® Probe RT-PCR Kit (Qiagen, Germantown, Maryland) on a CFX96™ detection module, combined with a C1000™ Touch Thermal Cycler (Bio-Rad Laboratories, Hercules, California). Each 25 µL qRT-PCR reaction included 12.5 µL 2× PCR Master Mix, 2 µL forward and reverse primers (10 µM) (Table 3), 0.5 µL probe (10 µM), 0.25 µL QuantiFast RTmix, 7 µL nuclease-free water and 1 µL template RNA. The qRT-PCR program began with a reverse transcription step at 50 °C for 10 min, followed with a PCR initial activation at 95 °C for 5 min, and finally 40 two-step cycles performed for 10 s at 95 °C; followed by 30 s at 60 °C.

The bioassay methods used for the adults were as described in the CONUS (Continental United States) manual for evaluating insecticide tolerance in *Cx. quinquefasciatus* mosquitoes using the CDC bottle bioassay kit. CDC provided technical grade malathion, CAS No. 121-75-5, with purity 98.2% (Chem Service, West Chester, Pennsylvania) and bioassay kits, including an aspirator, multiple 250-mL Wheaton® bottles with lids (Wheaton, Millville, New Jersey), and graduated disposable plastic pipettes. A stock solution and serial dilutions of malathion for the bioassays were prepared in acetone. The preliminary test was conducted to calculate LC_50_ of uninjected mosquitoes of the susceptible Sebring strain. Each bioassay in the preliminary test consisted of 10–25 six-day-old adults in the bottle coated with 1 mL malathion of five different concentrations (4 µg/mL, 8 µg/mL, 12 µg/mL, 100 µg/mL, and 400 µg/mL) previous diluted in acetone. The control bottle was treated with 1 mL of acetone. The mortality rate of tested mosquitoes was assessed after exposure to malathion for 120 min. The bioassay was repeated with at least three biological replicates. The LC_50_ was estimated using the dose-response curve on GraphPad Prism version 9.0.0 for Windows [58].

Three-day-old female adults were collected at random from the Sebring strain. Female mosquitoes were immobilized in the freezer at 4 ℃ for up to 10 min before dsRNA injection. Immobilized mosquitoes were placed on a Tissue-Tek® Cold Plate (Electron Microscopy Sciences, Hatfield, Pennsylvania). 300 nL of dsRNA (~ 300 ng) was injected into the thorax of the mosquito adults ventrally using a capillary needle. Capillary needles used for injection were pulled out using the Sutter Instrument® P-1000 Next Generation Micropipette Puller (Sutter Instrument, Novato, California). The program used in the needle puller was as follows: Heat = 458, Pull = 5, Velocity = 60, Delay = 255, Pressure = 500. The adults were collected after injection and reared under the normal insectary condition. Post-injected adults were fed 10% sugar water for three days and then collected for the CDC bottle bioassays to determine malathion resistance. Each experiment was repeated with at least three biological replicates.

The LC_50_ was measured and outcomes calculated according to the process described in the preliminary tests. Mosquito mortality was assessed after exposure to malathion for 120 min according to the criteria in the CONUS manual. The bioassay was repeated with at least three biological replicates. The significance analysis for relative gene expression was performed with the Student’s t test in R [53]. The significance analysis for mortality was performed with the Fisher’s LSD test using GraphPad Prism version 9.0.0 for Windows [58].

## Electronic supplementary material

Below is the link to the electronic supplementary material.

Supplementary Material 1

Supplementary Material 2

Supplementary Material 3

Supplementary Material 4

## Data Availability

The datasets generated and analyzed during the current study are available in the Gene Expression Omnibus (GEO) repository (GEO accession: GSE206489) at https://www.ncbi.nlm.nih.gov/geo/query/acc.cgi?acc=GSE206489 and from the first author (Cecilia.huang@ag.tamu.edu) upon request.

## List of abbreviations

P450s: Cytochrome P450s
GSTs: Glutathione S-transferases
DGE: Differential gene expression
RNA-Seq: High-throughput transcriptome sequencing
CDC: Centers for Disease Control and Prevention
MR: Malathion resistant sample
MS: Malathion susceptible sample
WI: Wild field-collected sample
CO: Laboratory-maintained susceptible Sebring strain
DEGs: Differentially expressed genes
dsRNA: Double-stranded RNA
RNAi: RNA interference
HCPH: Harris County Public Health
FE: Fold Enrichment
qRT-PCR: Real-time quantitative PCR
dsP450: Double-stranded RNA of cytochrome P450 gene
dsRFP: Double-stranded RNA of red fluorescent protein gene
GO: Gene Ontology
